# Allaying Post-COVID 19 Negative Health Impacts Among Older People: The “Need To Do Something With Others”—Lessons From the Japan Gerontological Evaluation Study

**DOI:** 10.1177/1010539520951396

**Published:** 2020-09-16

**Authors:** Miyako Kimura, Toshiyuki Ojima, Kazushige Ide, Katsunori Kondo

**Affiliations:** 1 St. Marianna University, Kanagawa, Japan; 2 Hamamatsu University School of Medicine, Shizuoka, Japan; 3 Chiba University, Chiba, Japan; 4 Hasegawa Hospital, Chiba, Japan; 5 National Center for Geriatrics and Gerontology, Aichi, Japan

## Background

Older people are considered the most vulnerable population in the current COVID-19 (coronavirus disease-2019) pandemic. While the negative impacts on health in the present are important, negative impacts on future health should also be considered. During the COVID-19 pandemic, older people have had more restrictions placed on them. This includes restrictions on going out, meeting other people, and participating in activities, all of which may negatively affect their social relationships.

The Japan Gerontological Evaluation Study (JAGES) has collaborated with more than 40 municipalities throughout Japan and has promoted evidence-based, gerontological research, targeting approximately 300 000 adults aged 65 years and older. To mitigate post-COVID-19 negative health impacts among older people, this article presents the results of JAGES, and discusses the importance of social relationships on the health of older people.

## Indirect Negative Health Impacts of COVID-19 on Older People

First, since the COVID-19 pandemic may increase social isolation among older people, opportunities for contact with others should be maintained, with special attention paid to frequency of contact (Table 1). According to Saito et al,^1^ older people who were in contact with others “from once a month to less than once a week” demonstrated a 1.40 times increase in the risk of functional disability, a 1.39 times increase in the risk of dementia, and a 1.15 times increase in the risk of premature death, when compared with individuals who reported they were in contact with others “frequently, every day (more than 9 per week).” Moreover, older people who were in contact with others “less than once a month” demonstrated a 1.37 times increase in the risk of functional disability, a 1.45 times increase in the risk of dementia, and a 1.34 times increase in the risk of premature death, when compared with individuals who were in contact with others “frequently, every day.” Similar results were also seen in a study by Aida et al.^2^ Therefore, to reduce the negative impact of COVID-19 on health among older people, contact with others at least once a week should be needed.

**Table 1.** Studies that Used Data From the JAGES (published 2011-2020)^a^.

Study	Years of collected data by JAGES	N (Analyze)	Outcomes	Adjusted variables	Explanatory variables	Results
Saito et al^1^	Followed-up from 2003 to 2013	12 085	Onset of functional disability, dementia, death	Age, gender, marital status, living status, educational attainment, annual equivalent income, disease, memory loss, living area	Social isolation (frequency of face-to-face, non-face-to-face contact with nonresident family members, relatives, and friends)	HRs for functional disability, dementia, and premature death (ref. contact with others more than 9 per a week): Contact with others “less than once a month”: adjusted HR: 1.37, 95% CI: 1.16-1.61, HR: 1.45, 95% CI: 1.21-1.74, and HR: 1.34, 95% CI: 1.16-1.55, respectively. Contact with others “from once a month to once a week” was also associated with these health indicators.
Aida et al^2^	Followed-up from 2003 to 2008	13 310	Mortality	Age, sex, BMI, self-rated health, present illness, smoking history, drinking, exercise, annual equivalent income, educational attainment	Cognitive social capital (general trust, social support, reciprocity) and structural social capital (social network: participation in political, industry, volunteer, citizen, religious, sports, neighborhood, avocation group, and frequency of meeting friends)	Low friendship network had significant association with high all-cause mortality. Male: meeting friends rarely (HR: 1.30, 95% CI: 1.10-1.53), female: having no friends (HR: 1.81, 95% CI: 1.02-3.23), after adjusting for covariates.
Tani et al^3^	Followed-up from 2010 to 2013	37 193	Depression	Age, education, equivalised household income, disease symptom, higher level of functional ability, frequency of vegitable or fruit intake, BMI, social support, social participation, frequency of meet friends, employment status, and marital status	Eating status, living status	The ARR for depression onset: Male: Those living alone and eating alone: ARR: 2.36 (95% CI: 1.18-4.71); those living with others and eating alone: ARR: 1.03 (95% CI: 0.81-1.32). Female: Those living alone and eating alone: ARR: 1.31 (95% CI: 1.00-1.72); those living with others and eating alone: ARR: 1.21 (95% CI: 1.01-1.44).
Nemoto et al^4^	Followed-up from 2003 to 2013	13 850	Dementia onset	Sex, age, educational attainment, marital status, living status, employment, drinking, smoking, walking time, IADLs, medical history (heart disease, stroke, hypertension, diabetes), and depression	Social participation (neighborhood associations/senior citizen clubs/fire-fighting teams, hobby groups, sports groups or clubs, political organizations or groups, industrial or trade associations, religious groups, volunteer groups, and citizen or consumer groups), position in the organization (leadership positions or regular members)	In young-old elderly (65-74 years), adjusted HR for dementia onset (ref. nonparticipants): Regular members or leadership positions: adjusted HR: 0.75: 95% CI: 0.64-0.88. Adjusted HR for dementia onset (ref. regular member): nonparticipants: adjusted HR: 1.22, 95% CI: 1.02-1.46; Leadership positions: HR: 0.81, 95% CI: 0.65-0.99.
Saito et al^5^	Followed for 3436 days (9.4 years) from 2003	13 984	Incident dementia	Sex, age, educational attainment, household income, depression, subjective cognitive impairment, IADL, walking time, stroke, diabetes, hobby	Social relationship: social networks (contact with friends, marital status), social support (social support exchange), social activity (participating in community group and engagement in paid work)	Being married, exchanging support with family members, having contact with friends, participating in community groups, and engaging in paid work were negatively related to incident dementia, after adjusting for covariates. The diversity scores (range 0-5) were associated with incident dementia (*P* < .001), and those who scored the highest were 46% less likely to develop incident dementia than those in the lowest category.
Tsuji et al^6^	Followed-up from 2010-2012 to 2016	40 308	Risk of cognitive impairment	Sex, age, disease status in treatment (stroke, hypertension, diabetes, hearing loss), depression, educational attainment, annual equivalent income, the presence of illnesses, depression, BMI, smoking, drinking, daily walking time, contact with others, frequency of meeting with friends/acquaintances, living status, population density, sunlight hours	Frequency of sports group participation	Higher prevalence of community-level sports group participation was associated with a lower risk of cognitive impairment (adjusted HR: 0.92; 95% CI: 0.86-0.99, estimated by 10 percentage points of participation proportion).
Fujihara et al^7^	Followed up from 2010-2012 to 2013	30 587	IADL	Sex, age, marital status, educational attainment, annual household income, the presence of illnesses, depression, BMI, smoking, drinking, daily walking time, frequency of going outside	Main predictor variable: Community-level social capital: civic participation (ie, participation in a volunteer group, a sports group, a hobby activity), social cohesion (ie, community trust and attachment), reciprocity (ie, receiving/providing emotional support or receiving instrumental support). Predictor variable: Individual-level social capital: participation in civic life, social cohesion, reciprocity.	Older people living in a community with higher civic participation presented significantly lower IADL disability (odds ratio: 0.90 per 1 standard deviation increase in civic participation score, 95% CI: 0.84-0.96), after adjusting for covariates.
Ide et al^8^	Followed -up for about 6 years from 2010	47 306	Incidence of functional decline	Age, sex, annual equivalent income, educational attainment, marital status, self-reported medical conditions, smoking, drinking, walking time, frequency of going outdoors, depression, emotional support, instrumental support, frequency of meeting friends, IADL	Social participation (neighborhood groups, hobby groups, sports groups or clubs, industrial groups, volunteer groups, and senior citizen clubs, work)	For rural and urban older people, participation in work (Rural: HR: 0.83; 95% CI: 0.76-0.91, urban: HR: 0.80; 95% CI: 0.70-0.91), participation in hobbies (Rural: HR: 0.76; 95% CI: 0.68-0.85, Urban: HR: 0.90; 95% CI: 0.84-0.97), and sports (Rural: HR: 0.79; 95% CI: 0.69-0.89, Urban: HR: 0.83; 95% CI: 0.77-0.91) was found to be protective against the incidence of decline, after adjusting for covariates.
Kanamori et al^9^	Followed-up from 2003 to 2007	11 581	Incident functional disability	Age, sex, annual equivalent income, educational attainment, marital status, occupational status, self-reported medical conditions, depression, smoking, drinking	Frequency of exercise (sports activities) and participation in sports organization	HRs for the incidence of functional disability (ref. active participation group, doing exercise once a month or more with participation in sport organization): Exercise alone group (doing exercise once a month or more without participation in sport organization): adjusted HR: 1.29, 95% CI: 1.02-1.64. Sedentary group (doing exercise less than once a month without participation in sport organization): adjusted HR: 1.65, 95% CI: 1.33-2.04.
Kanamori et al^10^	Followed-up from 2003 to 2007	12 951	Incident functional disability	Age, sex, annual equivalent income, educational attainment, marital status, occupational status, self-reported medical conditions	Social participation (neighborhood associations/senior citizen clubs/fire-fighting teams, hobby groups, sports groups or clubs, political organizations or groups, industrial or trade associations, religious organizations or groups, volunteer groups and citizen or consumer groups): the number of organizations for participations; types of the organizations	HRs for the incidence functional disability (ref. No participation): 1 participation: HR: 0.83, 95% CI: 0.73-0.95; 2 participation: HR: 0.72, 95% CI: 0.61-0.85; 3 or more participation: HR: 0.57, 95% CI: 0.46-0.70)
Takahashi et al^11^	Followed -up from 2003 to 2013	9741	The need for LTC or death at the end of the 9.4 years observational period, the incidence of the need for LTC or death at 2 and 5 years	Age, gender, living alone, educational attainment, smoking, drinking, walking time, annual household income, the number of comorbidities	Social participation (neighborhood associations/senior citizen clubs/fire-fighting teams, hobby groups, sports groups or clubs, political organizations or groups, industrial or trade associations, religious organizations or groups, volunteer groups, and citizen or consumer groups)	Social participation was strongly related to lower risk of the need for LTC (AOR: 0.82, 95% CI: 0.69-0.97) or death (AOR: 0.78, 95% CI: 0.70-0.88)

Abbreviations: JAGES, the Japan Gerontological Evaluation Study; HR, hazard ratio; CI, confidence interval; BMI, body mass index; ARR, adjusted rate ratio; IADL, instrumental activities of daily living, LTC, long-term care; AOR, adjusted odds ratio.

a In JAGES, we selected only larger studies (where the baseline survey included more than 10 000 participants) and those published after 2010 for the purpose of our study.

Second, while eating a healthy, balanced diet may be challenging during the COVID-19 pandemic, not only nutrition but eating status among older people should also be considered. According to Tani et al,^3^ compared with older males who ate with others, older males who ate alone and lived alone were 2.36 times more likely to demonstrate the onset of depression, while those who ate alone, but lived with others were 1.03 times more likely to develop depression. Similarly, compared with older females who ate with others, those who lived alone and ate alone were 1.31 times more likely to develop depression, while those who ate alone, but lived with others demonstrated a 1.21 times higher risk for the onset of depression.^3^ These results indicate that eating alone may increase the risk of depression, especially when combined with living alone, in older males. During the COVID-19 pandemic, older people living alone should receive special consideration, as they may face difficulties in going to restaurants or having lunches/dinners with others. Thus, the creation of opportunities to eat and communicate with others through virtual lunches and dinners should be considered.

Third, while social gatherings increase the risk of spreading the coronavirus, the benefits of social participation should also be taken into account. Using data from JAGES, various studies have presented the relationships between increased social participation and health, such as a decreased risk of dementia,^4,5^ and cognitive impairment,^6^ increased instrumental activities of daily living scores,^7^ decreased incidence of functional disability,^8-11^ and death.^11^ For example, Kanamori et al,^10^ found that older people who participated in one group demonstrated a 0.83 times lower incidence of disability, while those who participated in two groups demonstrated a 0.72 times, and those who participated in three or more different types of groups demonstrated a 0.57 times lower risk of disability than those who did not participate in any group. Similarly, Ide et al^8^ reported that the number of groups an older adult participated in was associated with lowered risk of functional decline in both rural and urban areas (0.76-0.92 times lower risks than those who did not participate in any group). Furthermore, compared with the active participant group (ie, exercising once a month or more, and participation in a sports organization), the sedentary group (ie, exercising less than once a month and no participation in a sports organization) exhibited a 1.65 times increase in risk, while the exercise-alone group (ie, exercising once a month or more and no participation in a sports organization) demonstrated a 1.29 times increased risk for incidence of functional disability.^9^ In other words, while regular exercise may reduce the incidence of functional disability, participation in a sports organization increases the preventive effects. Taken together, the above research suggests that participation in social activities is beneficial for health, and that if older people lose such opportunities for extended periods of time, their health may be negatively affected. Thus, the maintenance of social participation among older people during the pandemic is a critical health issue.

## Conclusions and Recommendations

The COVID-19 pandemic has restricted people’s lives, and its impact on health may be prolonged. This article has discussed the impact of social relationships on health among older people, based on cohort studies of JAGES, from a “super-aging” society. To ameliorate negative health impacts among older people post-COVID-19, these individuals should be encouraged to contact others, eat with others, and maintain organized social participation by practicing physical distancing. During mandated isolation, a minimum of weekly contact with others is recommended. Even non-face-to-face methods of contact could be beneficial, such as telephone, text, and/or video chat. Additionally, while eating with others and maintaining social participation in organized groups in-person may be difficult during a pandemic, participation could still occur via the internet. However, since internet use may be challenging for some older people, the construction of technological support networks may be needed. Avoiding pandemics such as COVID-19 may be impossible, but social relationships may help minimize the associated health risks in older individuals.
